# Lymphovascular or perineural invasion is associated with lymph node metastasis and survival outcomes in patients with gastric cancer

**DOI:** 10.1002/cam4.5701

**Published:** 2023-03-23

**Authors:** Fengxiang Zhang, Huaxian Chen, Dandong Luo, Zhizhong Xiong, Xianzhe Li, Shi Yin, Longyang Jin, Shi Chen, Junsheng Peng, Lei Lian

**Affiliations:** ^1^ Department of Gastrointestinal Surgery, Department of General Surgery, The Sixth Affiliated Hospital Sun Yat‐sen University Guangzhou China; ^2^ Guangdong Provincial Key Laboratory of Colorectal and Pelvic Floor Diseases, The Sixth Affiliated Hospital Sun Yat‐sen University Guangzhou China

**Keywords:** gastric cancer, lymph node metastasis, lymphovascular invasion, perineural invasion, prognosis

## Abstract

**Background:**

Lymphovascular invasion (LVI) and perineural invasion (PNI) are associated with poorer prognosis in several human malignancies, but their significance in gastric cancer (GC) remains to be clearly defined. Our study aimed to investigate the prognostic value of LVI/PNI in patients with curative resected GC.

**Methods:**

Records of 1488 patients with stage I‐–III GC and 3327 patients with stage I–III colorectal cancer (CRC) were reviewed retrospectively, and difference in the incidence of LVI/PNI between GC and CRC was compared. Univariate and multivariate analyses were used to evaluate whether LVI/PNI was an independent risk factor for lymph node metastasis (LNM) and overall survival (OS) in GC.

**Results:**

Patients with stage I–III GC had a significantly higher incidence of LVI/PNI than patients with stage I–III CRC (50.54% vs. 21.91%, *p*  < 0.001). LVI/PNI was significantly associated with higher CEA, higher CA199, deeper tumor invasion, more lymph node metastasis, and advanced TNM stage in GC ( *p*  < 0.05). Multivariate logistic regression analysis identified LVI/PNI (OR = 2.64, 95%CI: 2.05–3.40, *p*  < 0.001) as an independent risk factor for LNM in GC. The OS rate was significantly lower in the LVI/PNI‐positive GC group than that in the LVI/PNI‐negative GC group ( *p*  < 0.001). On multivariate Cox regression analysis, LVI/PNI (HR = 1.34, 95%CI: 1.04–1.71, *p*  = 0.023) was an independent prognostic factor for OS in GC.

**Conclusion:**

GC has a high incidence of LVI/PNI, which was closely associated with disease progression. LVI/PNI could serve as an independent risk factor for LNM and the prognosis of patients with curative resected GC. These findings will be helpful in predicting survival outcomes more accurately and establishing individualized treatment plans.

## Graphical abstract

Gastric cancer has a high incidence of LVI/PNI, which was closely associated with disease progression. LVI/PNI could serve as an independent risk factor for lymph node metastasis and the prognosis of patients with curative resected gastric cancer. These findings will be helpful in predicting survival outcomes more accurately and establishing individualized treatment plans.

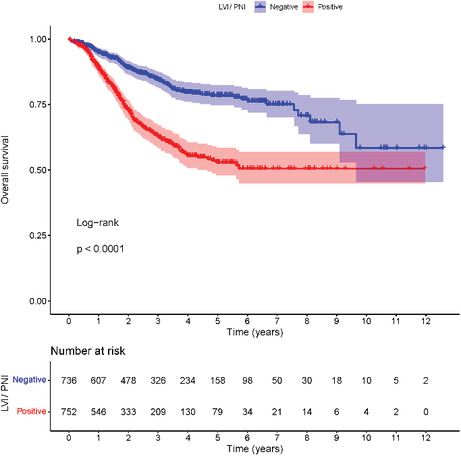

## 1 INTRODUCTION

Gastric cancer (GC) is the fifth most common malignancy and the fourth leading cause of cancer‐related deaths globally, which has the highest incidence and mortality rates in East Asian countries.^1^ GC is often diagnosed at an advanced stage with a poor prognosis, and lymph node metastasis (LNM) is an essential factor that negatively impacts the prognosis and determines clinical management for GC.^2^, ^3^, ^4^

Lymphovascular invasion (LVI) was histologically defined as the presence of tumor emboli within either the lymphatic or vascular channels or the destruction of the lymphatic or vascular wall by cancer cells.^5^ Perineural invasion (PNI) was histologically defined as tumor invasion of nerve structures and spread along nerve sheaths.^6^ LVI and PNI have been identified as a harbinger of poor prognosis for many malignancies; More interestingly, GC seems to have a relatively high incidence of LVI/PNI among various kinds of cancers, including colorectal cancer (CRC), esophageal squamous cell carcinoma, etc.^7^, ^8^, ^9^, ^10^, ^11^, ^12^, ^13^, ^14^ However, the prognostic value of LVI and PNI in GC is still under debate. Some researchers have found that LVI and PNI were independent risk factors for survival outcomes of patients with GC.^10^, ^15^, ^16^, ^17^, ^18^ Others found that LVI and PNI were not independent prognostic factors despite their strong association with disease progression in GC.^9^, ^19^, ^20^, ^21^, ^22^, ^23^, ^24^

We hypothesized that LVI/PNI is associated with LNM and survival outcomes in GC patients. In this study, we first compared the difference in the incidence of LVI/PNI between GC and CRC. Then, we analyzed associations between LVI/PNI and other clinicopathologic features in GC. Next, we identified clinicopathologic prognostic factors for lymph node metastasis. Finally, we evaluated the prognostic significance of LVI/PNI in GC patients who underwent curative gastrectomy.

## 2 METHODS

### 2.1 Patients

This is a retrospective study. Ethical Committee of The Sixth Affiliated Hospital, Sun Yat‐sen University, had approved this retrospective research, and the requirement for written informed consent was waived. Records of 2474 consecutive patients with GC who had surgical resection from January 2009 to May 2021 at The Sixth Affiliated Hospital, Sun Yat‐sen University were reviewed. The inclusion criteria were as follows: (1) primary adenocarcinoma of the stomach confirmed by histopathology; (2) pathologic stage I–III according to the criteria of the American Joint Committee on Cancer TNM staging system (seventh edition)^25^; (3) patients who underwent curative gastrectomy. Patients with incomplete clinicopathologic data, stage IV disease, noncurative operation, remnant GC, and patients who were lost to follow‐up were excluded. As a comparison, patients with pathologic stage I–III primary colorectal adenocarcinoma and complete LVI and PNI data between January 2017 and December 2018 were included.

Demographic and clinicopathologic characteristics were obtained from the Cancer Database of The Sixth Affiliated Hospital, Sun Yat‐sen University, including age (<60 years old, ≥60 years old), sex (male, female), body mass index (BMI) (<18.5 kg/m^2^, ≥18.5 kg/m^2^), preoperative hemoglobin (HGB) level (<90 g/L, ≥90 g/L), preoperative serum albumin level (<35 g/L, ≥35 g/L), preoperative carcinoembryonic antigen (CEA) level (<5 μg/L, ≥5 μg/L), preoperative carbohydrate antigen 125 (CA125) level (<35 U/mL, ≥35 U/mL), preoperative carbohydrate antigen 19–9 (CA199) level (<37 U/mL, ≥37 U/mL), tumor location (upper, middle, lower, or total), pathologic TNM stage, LVI, PNI, and most recent follow‐up data. Postoperative telephone follow‐up by the staff of the Cancer Database took place every 6 months up to 3 years, and annually thereafter. Preoperative laboratory tests were routinely performed for cancer patients, including HGB, serum albumin, and tumor markers. Gastrectomy resection specimens are routinely stained with hematoxylin and eosin (H&E) by Department of Pathology, and all specimens were analyzed by two experienced pathologists. LVI was considered positive when there were tumor emboli in lumina of endothelial‐lined spaces on H&E‐stained slides, and PNI was considered positive when tumor cells were found in perineural or intraneural spaces on H&E‐stained slides. In this study, LVI/PNI (+) means whether LVI or PNI was positive, and LVI/PNI (−) means both LVI and PNI were negative. Overall survival (OS) was calculated from the date of surgical resection to the date of last follow‐up or death for any cause.

### 2.2 Statistical analysis

Statistical analyses were performed using GraphPad Prism (version 8.0) and R software (version 4.0.2). Comparisons of categorical variables between groups were performed using the chi‐square test. Univariate and multivariate logistic regression analyses were used to identify prognostic factors for lymph node metastasis. Univariate and multivariate analyses of prognostic factors for OS in GC were performed using Cox proportional hazards models. Kaplan–Meier curves were generated for OS. Then differences in survival rates between groups were compared using the log‐rank test. All *p* values <0.05 were considered statistically significant.

## 3 RESULTS

### 3.1 Difference in the incidence of LVI/PNI between GC and CRC

**FIGURE 1:**
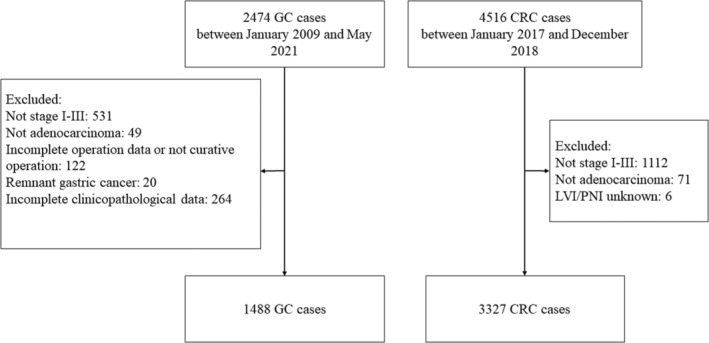
Flowchart of the study.

**FIGURE 2:**
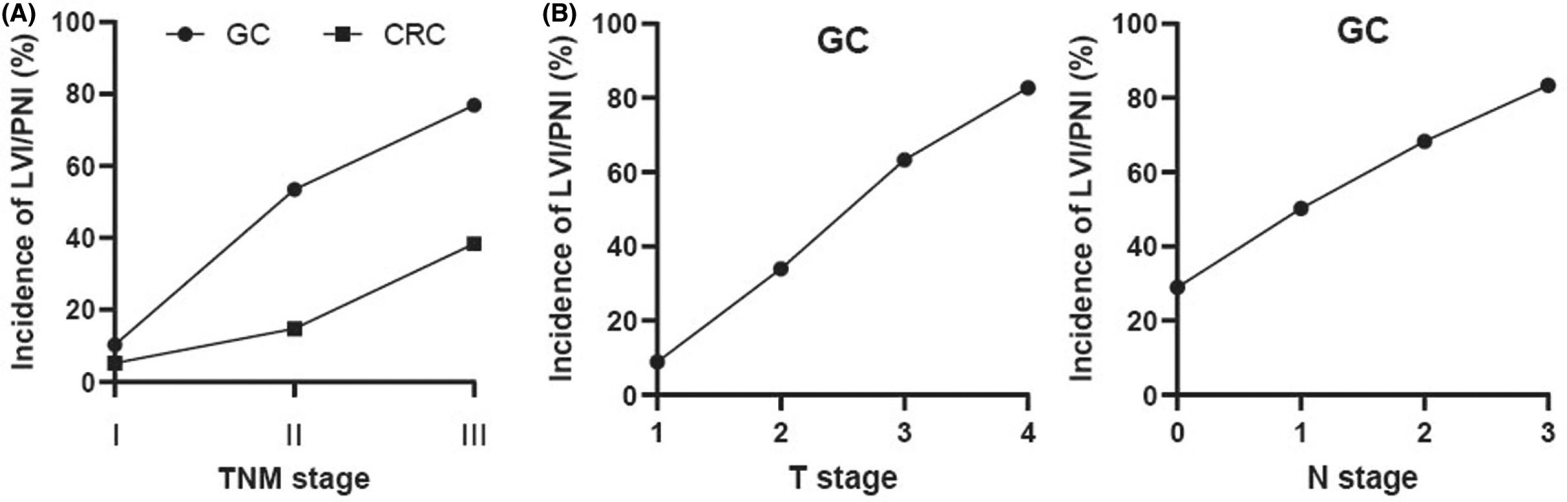
The incidence of LVI/PNI in gastric cancer (GC) and colorectal cancer. (A) Difference in the incidence of LVI/PNI between GC and colorectal cancer. (B) The incidence of LVI/PNI was increasing with advanced T stage or N stage in GC.

A total of 1488 patients with GC and 3327 patients with CRC were finally enrolled in this study, as shown in Figure 1. The incidences of LVI/PNI in both GC and CRC showed a growing tendency as the diseases progressed (Figure 2A). In detail, incidences of LVI/PNI in patients with stage I, II, and III GC were 10.32%, 53.47%, and 76.91%, respectively, while incidences of LVI/PNI in patients with stage I, II, and III CRC were 5.17%, 14.80%, and 38.41%, respectively. The GC patients had a significantly higher proportion of positive LVI/PNI than the CRC patients with the same pathologic TNM stage (*p* < 0.005). Besides, the overall incidence of LVI/PNI in stage I–III GC patients was also significantly higher than that in stage I–III CRC patients (50.54% vs. 21.91%, *p* < 0.001) (Table 1).

**TABLE 1:** Difference in the incidence of LVI/PNI between gastric cancer and colorectal cancer.

pTNM stage	Cancer	Overall	LVI/PNI		*p*‐value
			(+) (%)	(−) (%)	
I	Colorectal cancer	619	32 (5.17)	587 (94.83)	0.003
	Gastric cancer	407	42 (10.32)	365 (89.68)	
II	Colorectal cancer	1453	215 (14.80)	1238 (85.20)	<0.001
	Gastric cancer	518	277 (53.47)	241 (46.53)	
III	Colorectal cancer	1255	482 (38.41)	773 (61.59)	<0.001
	Gastric cancer	563	433 (76.91)	130 (23.09)	
I–III	Colorectal cancer	3327	729 (21.91)	2598 (78.09)	<0.001
	Gastric cancer	1488	752 (50.54)	736 (49.46)	

Abbreviations: LVI, lymphovascular invasion; PNI, perineural invasion.

### 3.2 Clinicopathologic features of stage I–III GC patients according to LVI/PNI status

Among the 1488 GC patients included in our study, 752 (50.54%) cases had LVI/PNI‐positive disease and 736 (49.46%) cases had LVI/PNI‐negative disease. As shown in Table 2, for GC patients with LVI/PNI‐positive disease, higher CEA level (*p* = 0.019), higher CA199 level (*p* < 0.001), deeper tumor invasion (*p* < 0.001), more LNM (*p* < 0.001), and advanced TNM stage (*p* < 0.001) were noted compared to those with LVI/PNI‐negative disease. Besides, the incidence of LVI/PNI was increasing in GC patients with advanced T stage or N stage (Figure 2B). There were no significant differences in terms of BMI, sex, age, HGB level, ALB level, CA125 level, and tumor location between the patients with and without LVI/PNI.

**TABLE 2:** Clinicopathologic characteristics of patients undergoing curative gastrectomy for stage I–III gastric cancer based on LVI/PNI status.

Characteristics	Overall *n* = 1488	LVI/PNI		*p*‐value
		(−) *n* = 736	(+) *n* = 752	
Sex (%)				
Male	1001 (67.27)	493 (66.98)	508 (67.55)	0.858
Female	487 (32.73)	243 (33.02)	244 (32.45)	
Age (years) (%)				
<60	688 (46.24)	335 (45.52)	353 (46.94)	0.618
≥60	800 (53.76)	401 (54.48)	399 (53.06)	
BMI (kg/m^2^) (%)				
<18.5	184 (12.37)	85 (11.55)	99 (13.16)	0.385
≥18.5	1304 (87.63)	651 (88.45)	653 (86.84)	
HGB (g/L) (%)				
<90	226 (15.19)	106 (14.40)	120 (15.96)	0.445
≥90	1262 (84.81)	630 (85.60)	632 (84.04)	
ALB (g/L) (%)				
<35	228 (15.32)	106 (14.40)	122 (16.22)	0.366
≥35	1260 (84.68)	630 (85.60)	630 (83.78)	
CEA (μg/L) (%)				
≤5	1247 (83.80)	634 (86.14)	613 (81.52)	0.019
>5	241 (16.20)	102 (13.86)	139 (18.48)	
CA199 (U/mL) (%)				
≤37	1294 (86.96)	665 (90.35)	629 (83.64)	<0.001
>37	194 (13.04)	71 (9.65)	123 (16.36)	
CA125 (U/mL) (%)				
≤35	1418 (95.30)	706 (95.92)	712 (94.68)	0.313
>35	70 (4.70)	30 (4.08)	40 (5.32)	
Tumor location (%)				
Total	12 (0.81)	5 (0.68)	7 (0.93)	0.838
Upper	429 (28.83)	211 (28.67)	218 (28.99)	
Middle	323 (21.71)	155 (21.06)	168 (22.34)	
Lower	724 (48.66)	365 (49.59)	359 (47.74)	
pT stage (%)				
T1	339 (22.78)	309 (41.98)	30 (3.99)	<0.001
T2	171 (11.49)	113 (15.35)	58 (7.71)	
T3	747 (50.20)	274 (37.23)	473 (62.90)	
T4	231 (15.52)	40 (5.43)	191 (25.40)	
pN stage (%)				
N0	642 (43.15)	456 (61.96)	186 (24.73)	<0.001
N1	295 (19.83)	147 (19.97)	148 (19.68)	
N2	274 (18.41)	87 (11.82)	187 (24.87)	
N3	277 (18.62)	46 (6.25)	231 (30.72)	
pTNM_stage (%)				
I	407 (27.35)	365 (49.59)	42 (5.59)	<0.001
II	518 (34.81)	241 (32.74)	277 (36.84)	
III	563 (37.84)	130 (17.66)	433 (57.58)	

Abbreviations: ALB, albumin; BMI, body mass index; CA, carbohydrate antigen; CEA, carcinoembryonic antigen; HGB, hemoglobin; LVI, lymphovascular invasion; PNI, perineural invasion.

### 3.3 Independent risk factors for lymph node metastasis in GC

As shown in Table 3, results of univariate logistic regression analysis showed that BMI, CEA level, CA199 level, T stage, and LVI/PNI status were associated with LNM in patients with GC (*p* < 0.05). In multivariate analysis, positive LVI/PNI (OR = 2.64, 95%CI: 2.05–3.40, *p* < 0.001) was identified as an independent risk factor for LNM in GC. Besides, lower BMI, higher CA199 level, and advanced T stage were also independent risk factors for LNM in GC.

**TABLE 3:** Results of univariate and multivariable logistic regression analyses for lymph node metastasis in gastric cancer.

Characteristics	Univariate analysis		Multivariate analysis	
	OR (95% CI)	*p* value	OR (95% CI)	*p*‐value
Sex				
Female	Reference			
Male	1.06 (0.85–1.32)	0.586		
Age (years)				
≥60	Reference			
<60	1.09 (0.89–1.34)	0.411		
BMI (kg/m^2^)				
≥18.5	Reference			
<18.5	1.62 (1.17–2.24)	0.004	1.50 (1.04–2.17)	0.030
HGB (g/L)				
≥90	Reference			
<90	1.31 (0.98–1.75)	0.069		
ALB (g/L)				
≥35	Reference			
<35	1.22 (0.92–1.63)	0.174		
CEA (μg/L)				
≤5	Reference			
>5	1.48 (1.11–1.97)	0.007	1.06 (0.77–1.47)	0.713
CA199 (U/mL)				
≤37	Reference			
>37	2.43 (1.73–3.41)	<0.001	1.50 (1.04–2.18)	0.032
CA125 (U/mL)				
≤35	Reference			
>35	1.58 (0.95–2.64)	0.077		
Tumor location				
Total	Reference			
Upper	1.55 (0.49–4.90)	0.452		
Middle	1.14 (0.36–3.61)	0.825		
Lower	1.28 (0.41–4.02)	0.668		
pT stage				
T1	Reference			
T2	2.90 (1.96–4.31)	<0.001	2.26 (1.51–3.40)	<0.001
T3	6.92 (5.14–9.32)	<0.001	4.04 (2.91–5.60)	<0.001
T4	18.14 (11.75–28.02)	<0.001	8.68 (5.42–13.91)	<0.001
LVI/PNI				
(−)	Reference			
(+)	4.96 (3.97–6.19)	<0.001	2.64 (2.05–3.40)	<0.001

Abbreviations: ALB, albumin; BMI, body mass index; CA, carbohydrate antigen; CEA, carcinoembryonic antigen; HGB, hemoglobin; LVI, lymphovascular invasion; PNI, perineural invasion.

### 3.4 Independent prognostic factors for survival outcomes in GC

**FIGURE 3:**
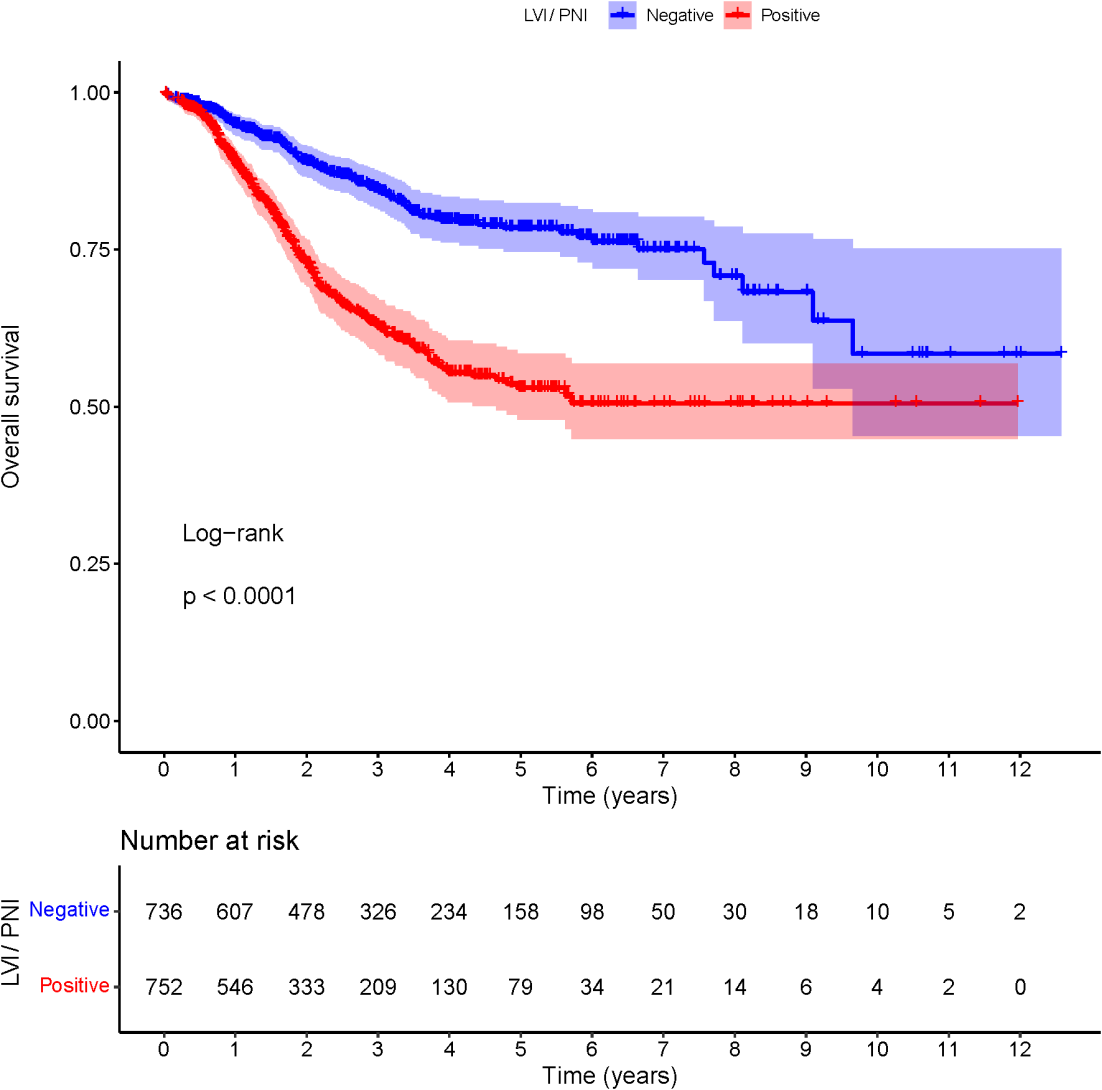
Kaplan–Meier analysis of overall survival based on LVI/PNI status among 1488 patients with stage I–III gastric cancer who underwent curative gastrectomy.

The median follow‐up time was 34.8 months. For GC patients with stage I–III disease, the 1‐year, 3‐year, and 5‐year OS rates in the LVI/PNI‐positive group were 88.9%, 62.8%, and 53.0%, respectively; and the 1‐year, 3‐year, and 5‐year OS rates in the LVI/PNI‐negative group were 94.9%, 84.3%, and 78.4%, respectively. The OS rate was significantly worse in the LVI/PNI‐positive group than that in the LVI/PNI‐negative group (*p* < 0.001) (Figure 3).

As shown in Table 4, univariate Cox regression analysis found that age, BMI, HGB, serum ALB level, CEA level, CA199 level, CA125 level, T stage, N stage, and LVI/PNI status were associated with OS of patients with stage I–III GC after curative resection (*p* < 0.05). Then multivariate analysis further identified positive LVI/PNI (HR = 1.34, 95%CI: 1.04–1.71, *p* = 0.023) as an independent prognostic factor for OS in GC patients. Besides, age, BMI, CA199 level, CA125 level, T stage, and N stage were also independent prognostic factors for OS of GC patients (*p* < 0.05).

**TABLE 4:** Results of univariate and multivariable Cox regression analyses for overall survival in gastric cancer.

Characteristics	Univariate analysis		Multivariate analysis	
	HR (95% CI)	*p* value	HR (95% CI)	*p*‐value
Sex				
Female	Reference			
Male	1.19 (0.94–1.50)	0.157		
Age (years)				
≥60	Reference			
<60	0.60 (0.48–0.75)	<0.001	0.64 (0.51–0.80)	<0.001
BMI (kg/m^2^)				
≥18.5	Reference			
<18.5	2.34 (1.81–3.02)	<0.001	1.89 (1.46–2.45)	<0.001
HGB (g/L)				
≥90	Reference			
<90	1.59 (1.22–2.07)	0.001	1.25 (0.94–1.65)	0.128
ALB (g/L)				
≥35	Reference			
<35	1.79 (1.37–2.34)	<0.001	1.26 (0.94–1.69)	0.117
CEA (μg/L)				
≤5	Reference			
>5	1.73 (1.35–2.22)	<0.001	1.24 (0.97–1.60)	0.091
CA199 (U/mL)				
≤37	Reference			
>37	2.29 (1.77–2.95)	<0.001	1.45 (1.11–1.88)	0.006
CA125 (U/mL				
≤35	Reference			
>35	2.52 (1.71–3.71)	<0.001	1.92 (1.29–2.86)	0.001
Tumor location				
Total	Reference			
Upper	1.22 (0.39–3.83)	0.736		
Middle	1.13 (0.35–3.57)	0.840		
Lower	0.90 (0.29–2.83)	0.860		
pT stage				
T1	Reference			
T2	2.55 (1.35–4.83)	0.004	1.80 (0.94–3.44)	0.076
T3	6.32 (3.85–10.35)	<0.001	3.37 (2.00–5.69)	<0.001
T4	10.92 (6.5–18.32)	<0.001	4.58 (2.59–8.07)	<0.001
pN stage				
N0	Reference			
N1	2.15 (1.56–2.96)	<0.001	1.63 (1.17–2.26)	0.004
N2	3.16 (2.32–4.29)	<0.001	1.94 (1.41–2.68)	<0.001
N3	4.73 (3.51–6.39)	<0.001	2.38 (1.71–3.31)	<0.001
LVI/PNI				
(−)	Reference			
(+)	2.50 (2.00–3.13)	<0.001	1.34 (1.04–1.71)	0.023

Abbreviations: ALB, albumin; BMI, body mass index; CA, carbohydrate antigen; CEA, carcinoembryonic antigen; HGB, hemoglobin; LVI, lymphovascular invasion; PNI, perineural invasion.

## 4 DISCUSSION

Gastric cancer is the fifth most common malignancy and the fourth leading cause of cancer‐related deaths worldwide.^1^ Previous studies have discussed the role of LVI/PNI in GC; however, the results are controversial and its prognostic value is still under debate. In the current research, we found that the incidence of LVI/PNI was significantly higher in GC compared with CRC. Our results revealed that LVI/PNI was associated with deeper tumor invasion and more lymph node metastasis. Moreover, we identified that LVI/PNI was an independent prognostic factor for survival outcomes in GC.

For many malignancies, LVI and PNI have emerged as important pathologic features and they have been identified as a harbinger of poor prognosis.^7^, ^8^, ^11^, ^12^, ^13^, ^14^ Interestingly, when we reviewed the literature, we found that in terms of LVI/PNI incidence, GC^9^, ^10^ seems to rank high among various kinds of cancers, including CRC,^7^, ^8^ esophageal squamous cell carcinoma,^11^ bladder cancer,^12^ prostate cancer.^13^, ^14^ The current study confirmed what we surmised. Our finding demonstrated that the GC patients did have a significantly higher proportion of positive LVI/PNI than the CRC patients with the same TNM stage. Moreover, incidences of LVI/PNI in GC patients with T4, N3, and stage III diseases were up to 82.68%, 83.39%, and 76.91%, respectively. This unusual phenomenon indicated that LVI/PNI might play a critical role in the development and progression of GC.

GC is often diagnosed at an advanced stage, and LNM is the major diffusion route that predicts a worse prognosis.^2^, ^3^, ^4^ However, the mechanism of LNM in GC is still not fully understood. In our study, 56.85% (846/1488) GC patients were diagnosed with lymph node metastasis, and incidences of LVI/PNI in patients with stage N0, N1, N2, and N3 GC were 28.97%, 50.17%, 68.25%, and 83.39%, respectively. Our study also found that 75.27% of the 752 patients with LVI/PNI had lymph node metastasis, and LVI/PNI was an independent risk factor for LNM in GC. One possible explanation is that the migration of cancer cells into vessels is an early step for nodal or distant metastasis.^26^ It is also reasonable to assume that the presence of LVI/PNI may indicate a more malignant phenotype with stronger invasiveness. Although the role of LVI/PNI in the occurrence of LNM is still unknown, our results suggest that occult metastasis should be suspected and close follow‐up should be considered when LVI/PNI is detected.

Prognostic value of LVI/PNI in GC is still under debate. Some researchers argued that LVI/PNI was not an independent prognostic factor despite its strong association with disease progression, including larger size of the neoplasm, deeper tumor invasion, more lymph node metastasis, and more distant metastasis.^9^, ^19^, ^20^, ^21^, ^22^, ^23^, ^24^ Conversely, other studies have identified LVI/PNI as an independent risk factor for survival outcome of patients with GC.^10^, ^15^, ^16^, ^17^, ^18^ Our results from the Kaplan–Meier analysis indicated that the OS rate was significantly worse in the LVI/PNI‐positive group than in the LVI/PNI‐negative group. Multivariate Cox regression model further identified LVI/PNI as an independent prognostic factor, which disproves the belief that the poor prognosis of the LVI/PNI‐positive GC patients is due to its close association with other established prognostic factors.

The major limitation of the current research is its design. It was a retrospective study from a single institution. Only patients with stage I–III GC who had curative gastrectomy were included in the study, and among them, patients with incomplete data were excluded. All of these can cause bias in the results. In this study, we aimed to demonstrate that GC had an unusually high incidence of LVI/PNI. Due to our limited research conditions, we only compared incidence of LVI/PNI of GC with that of CRC based on the Cancer Database of our hospital. As a supplement, we compared incidence of LVI/PNI of GC with that of other cancers reported by previous works of literature. And the collection time of GC patients and CRC patients was different. These factors might weaken the conclusion. In addition, chemotherapy regimen of patients, including data of neoadjuvant chemotherapy, could not be accurately tracked in the current cancer database and thus was not analyzed, which may have an impact on the survival outcomes in patients with advanced disease.

## 5 CONCLUSION

In conclusion, LVI/PNI is an underreported phenomenon in GC. Our study demonstrated that GC had a high incidence of LVI/PNI, which was closely associated with disease progression. LVI/PNI was identified as an independent risk factor for LNM and the prognosis of patients with stage I–III GC. These findings will be helpful to predict survival outcomes more accurately and establish individualized treatment plans.

## AUTHOR CONTRIBUTIONS

**Fengxiang Zhang:** Conceptualization (equal); data curation (equal); formal analysis (equal); investigation (equal); methodology (equal); project administration (equal); resources (equal); software (equal); validation (equal); visualization (equal); writing – original draft (equal); writing – review and editing (equal). **Huaxian Chen:** Conceptualization (equal); data curation (equal); formal analysis (equal); investigation (equal); methodology (equal); project administration (equal); resources (equal); software (equal); validation (equal); visualization (equal); writing – original draft (equal); writing – review and editing (equal). **Dandong Luo:** Conceptualization (equal); data curation (equal); formal analysis (equal); investigation (equal); methodology (equal); project administration (equal); resources (equal); software (equal); validation (equal); visualization (equal); writing – original draft (equal); writing – review and editing (equal). **Zhizhong Xiong:** Data curation (supporting); methodology (supporting); software (supporting); writing – review and editing (supporting). **Xianzhe Li:** Data curation (supporting); methodology (supporting); software (supporting); writing – review and editing (supporting). **Shi Yin:** Data curation (supporting); methodology (supporting); software (supporting); writing – review and editing (supporting). **Longyang Jin:** Data curation (supporting); methodology (supporting); software (supporting); writing – review and editing (supporting). **Shi Chen:** Conceptualization (equal); funding acquisition (equal); resources (equal); supervision (equal). **Junsheng Peng:** Conceptualization (equal); funding acquisition (equal); resources (equal); supervision (equal). **Lei Lian:** Conceptualization (equal); funding acquisition (equal); resources (equal); supervision (equal).

## FUNDING INFORMATION

This study was supported by the Guangdong Natural Science Fund for Outstanding Youth Scholars (Grant No. 2020B151502067) and National Key Clinical Discipline.

## CONFLICT OF INTEREST STATEMENT

The authors have no conflict of interest regarding the content of the article.

## ETHICS STATEMENT

The current research complies with the standards of the Declaration of Helsinki. Ethical Committee of The Sixth Affiliated Hospital, Sun Yat‐sen University had approved this retrospective research (No. 2022ZSLYEC‐232), and the requirement for written informed consent was waived.

## Data Availability

All analyzed data are included in this article. The original data are available upon reasonable request to the corresponding author.
